# Response to letter to the editor: “comment on unplanned out-of-hospital birth and risk factors of adverse perinatal outcome: findings from a prospective cohort”

**DOI:** 10.1186/s13049-019-0635-1

**Published:** 2019-05-28

**Authors:** François Javaudin, Arnaud Legrand, Philippe Pes, Emmanuel Montassier, Christelle Volteau

**Affiliations:** 1 Department of Emergency Medicine, CHU Nantes, Nantes, France; 2 MiHAR lab, Université de Nantes, 44000 Nantes, France; 3 DRCI, CHU Nantes, Nantes, France

**Keywords:** Methodology, Multiple logistic regression, Firth method, Bootstrapping, Predictive performance

## Abstract

The aim of this Letter to the Editor was to respond to a comment highlighting potential statistical biases in an analysis of our recently published article. We therefore specified the method for selecting the model variables in order to limit overfitting, then we used the Firth method to control the sparse data bias, and finally for checking internal validity we used bootstrapping methods. In total, the conclusions of our model were not changed by these new analyses.

To the Editor:

We thank Dr. Yarandi and Tehrani [1] for their interest in our study [2] and to challenge us on specific statistical aspects. To avoid misinterpretations, we will try to clarify methodological trade-off we consent, and reveal complementary details and information of our dataset deep analysis.

Firstly, to limit overfitting, even if it was not explicitly described in the statistical part of the original paper, we indeed select predictor variables to input our multiple logistic regression model, on the basis of a stringent *p*-value < 0.2. Looking at Table 2 of our original research, we can see that the selected variables defending a p-value between 0.03 and < 0.0001.

Secondarily, we admit that we didn’t process any penalization methods to control sparse data biases [3] on multiple pregnancy. But when we apply Firth method [4], model is strictly conserved and extremely wide interval remains tangible: Odds Ratio = 47.9; Confidence Interval 95%: 3.5–663.6. Moreover, even if multiple pregnancy, hypothermia, and/or materno-foetal infection can lead to circularity over-risk due to the imbrication of these clinical contexts with prematurity status, we rigorously explored intrinsic contribution and correlation and covariance matrices. For example, Pearson chi2 test between “Prematurity” and “Hypothermia” highlights their independent predictive factor status (*p*-value = 0.20).

Finally, the predictive performance of model was internally validated through bootstrapping [5]. The multivariate model was refitted in 200 bootstraps samples (samples of the same size as the original population but with patients drawn randomly with replacement from the full study population) and then tested on the original sample. The measure of predictive discrimination used to characterize model performance, in both the original sample and the validation samples, was the area under the receiver operating characteristic curve (AUC). Average optimism, as the difference between bootstrap performance and test performance, was calculated. Optimism-corrected performance was then estimate by subtraction of the average optimism from the apparent performance (initial multivariate model). SAS software version 9.4 was used for validation. Initial multivariate model had an AUC of 0.78. In the 200 bootstrap samples, the mean AUC was 0.80. When the models from each bootstrap sample were tested in the original sample, the AUC decreased to 0.75. The optimism was then equal to 0.05 and the optimism-corrected AUC equal to 0.73.

In total, despite the potential biases you mentioned, these results confirm our multivariate model as a first step exploratory analysis on morbi-mortality determinants in unplanned out-of-hospital birth.

## Abbreviation

AUC: Area under the receiver operating characteristic curve
